# Incidence, risk factors and impact of seasonal influenza in pregnancy: A national cohort study

**DOI:** 10.1371/journal.pone.0244986

**Published:** 2021-01-15

**Authors:** Nicola Vousden, Kathryn Bunch, Marian Knight, the UKOSS Influenza Co-Investigators Group

**Affiliations:** 1 School of Population Health & Environmental Sciences, Faculty of Life Sciences & Medicine, King's College London, London, United Kingdom; 2 Policy Research Unit in Maternal Health and Care, National Perinatal Epidemiology Unit, University of Oxford, Oxford, United Kingdom; Bradford Institute for Health Research, UNITED KINGDOM

## Abstract

**Background:**

Pregnant women are particularly vulnerable to severe infection from influenza resulting in poor neonatal outcomes. The majority of evidence relates to pandemic 2009 A/H1N1 influenza. The objective of this study was to describe the characteristics and outcomes of pregnant women hospitalised with seasonal influenza.

**Methods:**

This national, prospective, observational cohort study used the UK Obstetric Surveillance System (UKOSS) to identify all pregnant women admitted to hospital between 01/11/2016 and 31/10/2018 with laboratory confirmed influenza at any gestation and up to two days after giving birth. These were compared to women admitted to give birth that did not have influenza. Baseline characteristics, immunization status, maternal and perinatal outcomes were compared.

**Results:**

There were 405 women admitted to hospital with laboratory confirmed influenza in pregnancy: 2.7 per 10,000 maternities. Compared to 694 comparison women, women with influenza were less likely to be professionally employed (aOR 0.59, 95%CI 0.39–0.89) or immunised in the relevant season (aOR 0·59, 0·39–0·89) and more likely to have asthma (aOR 2.42, 1.30–4.49) or have had a previous pregnancy complication (aOR 2·47, 1·33–4·61). They were more likely to be admitted to intensive care (aOR 21.3, 2.78–163.1) and to have a cesarean birth (aOR 1·42, 1·02–1.98). Their babies were more likely to be admitted to neonatal intensive care (aOR 1.86, 1·01–3·42).

**Conclusions:**

Immunization reduces the risk of hospitalisation with influenza in pregnancy which is associated with increased risk of morbidity for both the mother and baby. There is a continued need to increase awareness of safety and effectiveness of immunization in pregnancy and provision within antenatal care settings, especially for high-risk groups.

## Introduction

In 2012, the World Health Organization recommended that countries initiating or expanding seasonal influenza immunization programmes should prioritise pregnant women over other high-risk groups [1]. In the UK, national guidance since 2010–11 has been that all pregnant women, at any gestation, should receive immunization, with an ambition to reach at least 75% of this population [2]. This policy is based on increased risk of complicated influenza in pregnant women, protection of both mother and infant and the safety and effectiveness of the vaccine [1], yet uptake varies globally [3].

Confirmed seasonal influenza is reported to affect between 483 to 1097 pregnant women per 10,000 and between 3 to 91 infants per 10,000 [4, 5]. Evidence, predominantly from the pandemic influenza period in 2009 (H1N1), has demonstrated that pregnant women were particularly vulnerable to severe infection [6–10], which results in increased risk of hospital admission [11]. Recent systematic analyses, which take account of important confounders such as prior vaccine exposure and age, have challenged the widespread belief that pregnant women are at greater risk of mortality and severe morbidity from influenza [11, 12]. While pregnant women are more likely to be hospitalised, the likelihood of maternal mortality or intensive care admission are not increased compared to the general population of women of reproductive age [11]. However, the quality of evidence to date is very low and preventable deaths from influenza are still reported [13].

Factors associated with increased risk of hospital admission after pandemic influenza infection in pregnancy include maternal obesity, asthma, multiparity, multiple pregnancy, black or other minority group ethnicity and smoking among women younger than 25 years [6, 8, 10, 14]. Pandemic influenza has also been widely reported to increase the risk of poor infant outcome such as preterm birth and stillbirth [11], although more recent studies suggest this is only observed in women with underlying medical comorbidity [15] and is not observed in cases of mild influenza [16].

Recent evidence suggests that the increased risk of hospitalisation may also be observed in pregnant women with seasonal influenza [17, 18], which again is more likely in women of non-European ethnicity [17], and women with medical co-morbidities such as asthma [19]. However, the lack of high-quality studies reporting seasonal influenza makes it challenging to assess the risk of associated morbidity, the characteristics of those at risk or to compare this risk to pandemic influenza and inform associated policy. Recent systematic reviews have concluded that further evidence regarding the impact of seasonal influenza on maternal and neonatal outcomes is required [11, 20].

Influenza immunization during pregnancy is widely reported to have no negative impact on pregnancy outcomes. Benefits in terms of reduction in risk of certain adverse pregnancy outcomes, including preterm birth [21, 22], low birth weight [22, 23] and stillbirth [24], have been reported. Immunization is effective at preventing seasonal influenza in pregnant women and their infants in both high and low-resource settings [25–28] and is cost-effective for both seasonal and pandemic influenza [29–31]. Despite this, uptake in pregnant women is low, with 45% of all pregnant women in England receiving immunization in 2018–19, a reduction from 47% the year before [32]. Monitoring vaccine uptake in pregnant women is complicated by the dynamic nature of the group, coding for pregnancy status and differing place of vaccine administration, with immunization available in primary care and some community pharmacies as well as antenatal clinics [32]. This may contribute to an underestimation of reach, but it is likely this still falls considerably short of the overall national target of 75% coverage of risk groups in the UK.

The most commonly cited reasons for not being immunised include perceptions of risk of influenza and concern over effectiveness or safety of the immunization [33–35]. Characteristics of women associated with an increased likelihood of receiving seasonal influenza immunization are White British ethnicity [35], professional employment and education [33, 36], immunization in a previous pregnancy and receiving information around immunization from a healthcare professional [36]. Similar characteristics were associated with uptake of pandemic flu vaccine in 2009 [37, 38].

There is thus clear evidence that pregnant women are at greater risk of being hospitalised with influenza and that pandemic influenza may negatively impact on pregnancy outcomes. Seasonal influenza immunization during pregnancy protects against influenza in women and their children and is safe and cost-effective. However, there is insufficient evidence to describe the impact of seasonal influenza on pregnancy outcomes, the characteristics of those at greatest risk and therefore the associated influence of prior immunization. These data will inform policy and investment at a national and international level.

The objectives of this study were to:

- Use a national maternity research platform to determine the incidence of hospitalisation with seasonal influenza in pregnancy in the UK.
- Identify the characteristics associated with hospitalisation with seasonal influenza in pregnancy.
- Determine the impact of severe seasonal influenza in pregnancy on outcomes for mother and infant.
- Identify the characteristics associated with immunization for seasonal influenza in pregnancy.
- Determine the impact of immunization on maternal and infant outcomes.

## Materials and methods

**Fig 1:**
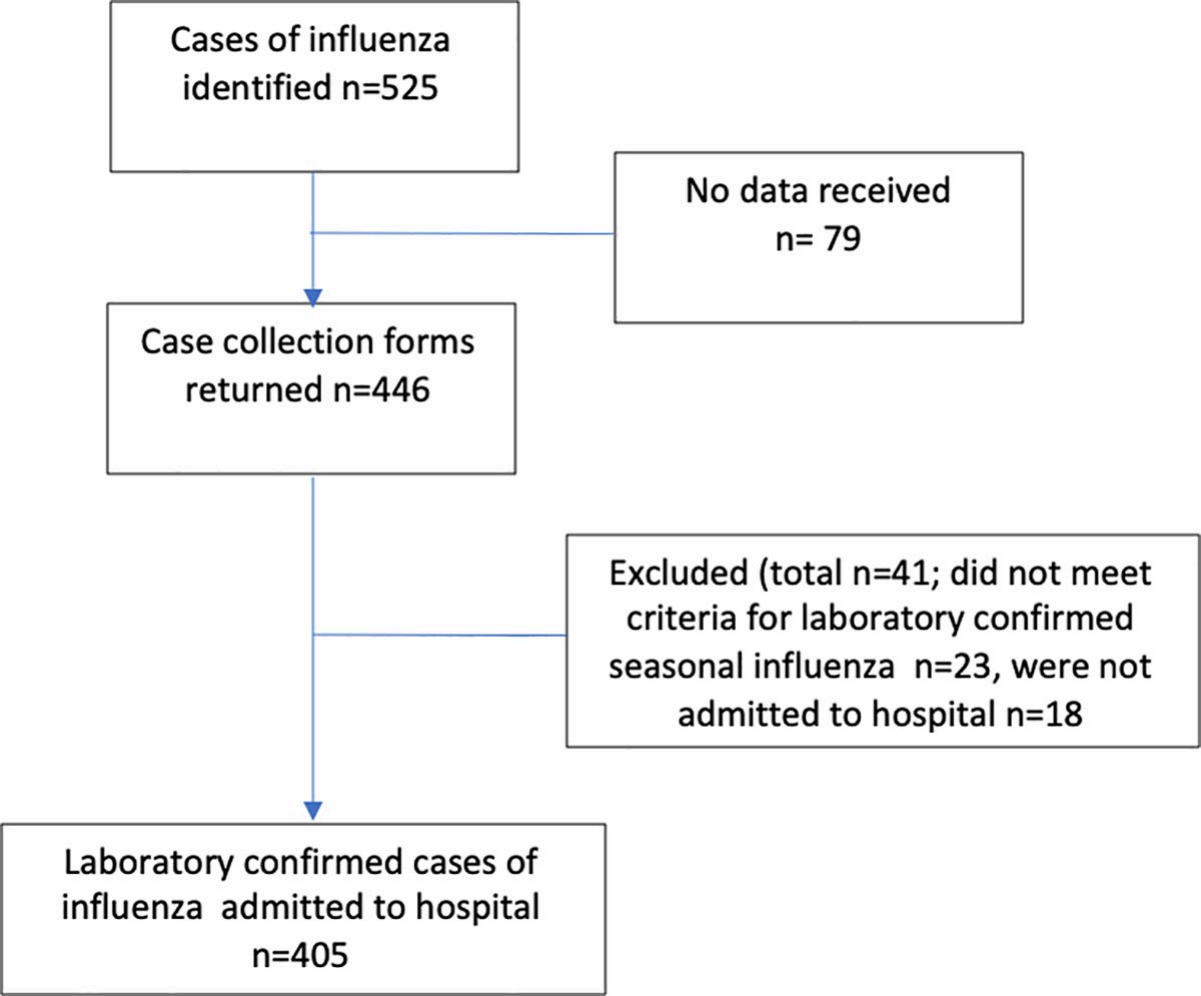
Identification of case women eligible for inclusion in the study.

A national, prospective, observational cohort study was undertaken using UK Obstetric Surveillance System (UKOSS). UKOSS is a national research platform that includes all 194 NHS consultant‐led obstetric units in the UK and has been established since 2005 [39]. Therefore, all women who are admitted to a hospital in the UK during pregnancy or in the immediate postnatal period will be captured. Each unit in the UK has a nominated UKOSS clinician data collector who are sent a monthly notification email containing a list of conditions under surveillance and are asked to report the number of women with these conditions or to confirm zero cases. In this study, the case definition included all women in the UK admitted to hospital between 1st November 2016 and 30th October 2018 with influenza infection confirmed on virological testing, at any stage of pregnancy or up to two days after delivery. On reporting a woman with influenza, the clinician completed a data collection form with anonymised data extracted from medical records. If forms with complete data were not returned, up to five email reminders were sent. Women were excluded if a data collection form was not returned (n = 79) or they did not meet the definition of laboratory confirmed influenza during admission to hospital whilst pregnant (n = 41) (Fig 1).

For each woman data were collected about diagnosis and management, as well as maternal demographics, immunization status, obstetric and medical history. Pregnancy outcomes including admission to intensive care and the occurrence of major maternal morbidities (composite including cardiac arrest, cerebrovascular accident, adult respiratory distress syndrome, disseminated intravascular coagulopathy, HELLP, pulmonary oedema, secondary infection, renal failure, thrombotic event, septicaemia, required ventilation) were collected. As were delivery and perinatal outcomes including preterm delivery, stillbirth and admission to neonatal intensive care.

Information on women who died from influenza in pregnancy, or consequent stillbirths or neonatal deaths, was cross-checked with data from the MBRRACE-UK: Mothers and Babies: Reducing Risk through Audits and Confidential Enquiries across the UK collaboration, the organisation responsible for maternal and perinatal death surveillance in the UK. If any additional women were identified through these sources which had not been identified through UKOSS, the nominated UKOSS clinician in the relevant hospital was contacted and asked to provide further information on management and outcomes.

Since all consultant led obstetric units report to UKOSS, this system captures all women hospitalised with influenza from the entire pregnancy population. Therefore, the study sample size was governed by the disease incidence in the population and no formal power calculation was carried out. The incidence of hospitalisation with seasonal influenza was thus calculated using denominators from national maternity data (2018) for the constituent countries of the United Kingdom.

The comparison group consisted of the two women that delivered in the same hospital immediately before each woman diagnosed with influenza between 1^st^ November 2017 and 30^th^ October 2018. Delivery suite records were used to identify this group by using the date and time of delivery for the woman who was hospitalised with confirmed influenza and then identifying the two women that gave birth immediately before this woman, whom did not have influenza. Records were unavailable for 116 comparison women.

### Ethics committee approval

This study was approved by the North London REC1 (REC Ref. Number: 10/H0717/20). Participant consent for collection of anonymised data from patient records was not required.

### Statistical analysis

The incidence of seasonal influenza was calculated using denominators from national maternity data from 2016, 2017 and 2018 for the United Kingdom. A maternity was defined as a woman giving birth to one or more children including stillborn babies at 24 weeks’ gestation or greater. Baseline characteristics were compared between women hospitalised with seasonal influenza and comparison women without seasonal influenza using univariate logistic regression. Maternal and perinatal outcomes were compared between women with seasonal influenza and those without seasonal influenza and adjusted for potential confounders as identified through the univariate analysis (Asthma requiring regular steroid treatment, Immunization status, Employment status, and relevant previous pregnancy problems), using multivariable unconditional logistic regression. An exploratory analysis was undertaken to investigate whether outcomes in women hospitalized with influenza differed according to immunisation status. A sub-analysis was undertaken to look at the characteristics associated with immunization within the comparison group who did not have seasonal influenza. Potential confounders identified during the univariate analysis (smoking status and estimated date of delivery) were adjusted of in multivariable unconditional logistic regression. Cases with confirmed seasonal influenza were not included in the analysis of immunization status as by nature of having influenza, they may not be representative of the general population.

Proportions, adjusted and unadjusted odds ratios are presented with 95% confidence intervals. Where data were missing, proportions are presented out of cases known. Statistical analyses were undertaken using STATA version 15.

## Results

In total, there were 405 women admitted to hospital with laboratory confirmed influenza in pregnancy (exposed cohort) during the study (Fig 1). Information was received for 694 unexposed comparison women (86% of those requested). There were 1,476,405 maternities during this period, therefore the overall incidence of confirmed influenza requiring hospital admission in pregnancy was 2.7 per 10,000 maternities (95% CI 2.5–3.0).

Women hospitalised with seasonal influenza in pregnancy were less likely to be professionally employed (15% versus 21%; aOR 0.59, 0.39–0.89) and less likely to be immunised in the relevant season than comparison women without seasonal influenza (26% versus 31%; aOR 0·62, 0·45–0·86) (Table 1). Women hospitalised with influenza were more likely to have asthma (10% versus 4%; aOR 2.42, 1.30–4.49), to have a raised body mass index (29% versus 22% OR 1·37, 1·04–1·82) and to have had a previous pregnancy complication than those in the comparison group (11% versus 5%; aOR 2·47, 1·33–4·61). There was no difference in age, marital status, smoking, parity, multiple pregnancy or pre-existing medical conditions other than asthma in women admitted to hospital with seasonal influenza compared to those without seasonal influenza.

**Table 1:** Characteristics of case and comparison women.

Independent Variables	Number (%) of cases (n = 405)	Number (%) of comparison women (n = 694)	OR (95% CI, *P*-value)	aOR^a^ (95% CI, *P*-value)
**Maternal age (years)**				
<20	14 (3)	18 (3)	1·30 (0·64–2·65, p = 0.475)	-
20–35	286 (71)	477 (69)	1.00 (-)	-
≥ 35	105 (26)	199 (29)	0·88 (0·67–1·16, p = 0.368)	-
**Socioeconomic status**				
Professional	61 (15)	143 (21)	0·72 (0·51–1·01, p = 0.003)	0·59 (0·39–0·89, p = 0.013)
Non-professional employed	233 (58)	394 (57)	1·00 (-)	1·00 (-)
Unemployed	64 (16)	76 (11)	1·42 (0·98–2·06, p = 0.061)	1·23 (0·79–1·92, p = 0.354)
Unknown	47 (12)	81 (12)		-
**Marital Status**				
Married/Cohabiting	327 (81)	588 (85)	1·00 (-)	-
Single	76 (19)	96 (14)	1·42 (1·02–1·98, p = 0.036)	-
Unknown	2 (0)	10 (1)		
**Smoking Status during pregnancy**				
Did not smoke	317 (78)	549 (79)	1·00 (-)	-
Smoked	86 (21)	135 (19)	1·10 (0·81–1·49, p = 0.526)	-
Unknown	2 (0)	10 (1)		
**Parity**				
Nulliparous	129 (32)	274 (39)	0·72 (0·55–0·93, p = 0.011)	-
Multiparous	276 (68)	420(61)	1·00 (-)	-
**Body Mass Index**				
Normal or overweight (<30 kg/m^2^)	282 (70)	518 (75)	1·00 (-)	-
Obese (≥30 kg/m^2^)	116 (29)	155 (22)	1·37 (1·04–1·82, p = 0.026)	-
Unknown	7 (2)	21 (3)		
**Multiple Pregnancy**				
Yes	12 (3)	13 (2)	1·60 (0·72–3·54, p = 0.246)	-
No	393 (97)	681 (98)	1·00 (-)	-
**Pre-existing medical condition**				
Yes	56 (14)	77 (11)	1·29 (0·89–1·86, p = 0.182)	-
No	348 (86)	615 (89)	1·00 (-)	-
Unknown	1 (0)	2 (0)		
**Pre-existing Asthma**				
Yes	41 (10)	30 (4)	2·50 (1·53–4.07, p = 0.041)	2·42 (1·30–4·49, p = 0.005)
No	363 (90)	663 (96)	1·00 (-)	1·00 (-)
Unknown	1 (0)	1 (0)		
**Known previous pregnancy complications**				
Yes	43 (11)	35 (5)	2·24 (1·41–3·56, p = 0.001)	2·47 (1·33–4·61, p = 0.004)
No	362 (89)	659 (95)	1·00 (-)	1·00 (-)
**Immunization Status**				
Ever Immunised against influenza				
Yes	137 (34)	267 (38)	0·69 (0·53–0·92, p = 0.010)	-
No	190 (47)	257 (37)	1·00 (-)	-
Unknown	78 (19)	170 (25)		
Immunised in the relevant influenza season				
Yes	106 (26)	214 (31)	0·65 (0·48–0·87, p = 0.004)	0·62 (0·45–0·86, p = 0.004)
No	202 (50)	264 (38)	1·00 (-)	1·00 (-)
Unknown	97 (24)	216 (31)		

^a^OR adjusted for Asthma requiring regular steroid treatment, Immunization status, Employment status, and relevant previous pregnancy problems.

Three percent of women (n = 13) were admitted with flu in the first trimester, compared to 18% (n = 70) in the second and 79% (n = 309) third trimester. Of the 405 women with influenza, 308 (76%) had no specific influenza type recorded. Of the 97 women with a recorded strain, 17 (18%) had A/H1N1, 73 (75%) had influenza A/H3, 6 (6%) had other influenza A strains and only 1 (1%) had a non-A flu strain. The majority of women admitted with influenza received antiviral agents (n = 349, 86%) with oseltamivir (Tamiflu) being most commonly used (n = 348, 99.7%). The median duration of hospital admission was 3 days (IQR 2-5days), noting that for some women this included a postnatal stay potentially unrelated to their influenza.

Maternal and perinatal outcomes are shown in Table 2. Twenty-one women with influenza (5%) were admitted to intensive care (ITU) as opposed to one woman in the comparison group of women without influenza (aOR 21·3, 2·78–163·1), with a median admission of 3 days (IQR 2–6) compared to 5 days for the comparison woman’s ITU admission. Only one woman admitted with influenza received extra-corporeal membrane oxygenation (ECMO). Just over a quarter of women with influenza gave birth during the same admission as their influenza admission (n = 114, 28%). Women with influenza were more likely to have a cesarean birth (aOR 1·42, 1·02–1·98). There was a statistically non-significant increased risk of maternal morbidity in women with influenza compared to comparison women without influenza (aOR 2·23, 0·78–6·43). There were no increased odds of pregnancy loss or assisted vaginal birth in women admitted to hospital with seasonal influenza compared to those without seasonal influenza.

**Table 2:** Maternal and perinatal outcomes.

	Cases (n = 405)	Comparison women (n = 694)	OR (95% CI, *P*-value)	aOR^a^ (95% CI, *P*-value)
	Number (% of known)	Number (% of known)		
***Maternal Outcomes***				
Admission to ITU	21 (5)	1 (0)	37·9 (5·08–283·2, p = <0.001)	21.3 (2.78–163.1, p = 0.003)
Delivered at time of influenza admission	114 (28)			
Pregnancy loss	5 (1)	2 (0)	4·33 (0·84–22·4, p = 0.081)	3·86 (0·39–37.9, p = 0.247)
Maternal Morbidity^b^	13 (3)	11 (2)	2·08 (0·92–4·69, p = 0.077)	2·23 (0·78–6·43, p = 0.137)
Induction of Labour	167 (43)	240 (35)	1·42 (1·10–1·83, p = 0.007)	1·21 (0·87–1·66, p = 0.253)
**Mode of delivery**^**c**^				
Cesarean	151 (38)	200 (29)	1·56 (1·20–2·03, p = 0.001)	1·42 (1·02–1.98, p = 0.040)
Emergency caesarean	76 (19)	90 (13)	1·62 (1·16–2·27, p = 0.005)	1·42 (0·92–2·18, p = 0.110)
Assisted vaginal delivery	33 (8)	71 (10)	0·81 (0·52–1·25, p = 0.336)	0·95 (0·55–1·63, p = 0.853)
***Perinatal Outcome***				
Preterm Delivery <37 weeks	52 (13)	55 (8)	1·77 (1·19–2·65, p = 0.005)	1·60 (0·94–2·73, p = 0.083)
Very Preterm Delivery <32 weeks	7 (2)	11 (2)	1·13 (0·43–2·93, p = 0.808)	0·65 (0·16–2·64, p = 0.550)
Live birth	385 (99)	689 (100)	0·22 (0·04–1·16, p = 0.074)	0·14 (0·01–1·34, p = 0.088)
Stillbirth/Perinatal death	5 (1)	3 (0)	2·98 (0·71–12·5, p = 0.137)	3·89 (0·64–23.4, p = 0.139)
Admission to neonatal unit	40 (10)	32 (5)	2·39 (1·47–3·87, p = <0.001)	1·86 (1·01–3·42, p = 0.048)
Congenital abnormality	5 (1)	10 (1)	0·89 (0·30–2·63, p = 0.838)	0·82 (0·21–3·26, p = 0.779)
Low birth weight (<10^th^ centile)	27 (7)	56 (8)	0·85 (0·53–1·37, p = 0.507)	0.95 (0·53–1·70, p = 0.853)
Gestation at delivery (weeks), Median (IQR)	39 (38–40)	39 (38–40)		

^a^OR adjusted for Asthma requiring regular steroid treatment, Immunization status, Employment status, and relevant previous pregnancy problems

^b^Composite of major medical complications including: cardiac arrest, cerebrovascular accident, adult respiratory distress syndrome, disseminated intravascular coagulopathy, HELLP, pulmonary oedema, secondary infection, renal failure, thrombotic event, septicaemia, required ventilation.

^c^In women over 24 weeks of gestation (excludes five cases and two comparison women).

The median gestation at delivery was 39 weeks both for women hospitalised with influenza and for the comparison women. A higher proportion of women with influenza gave birth at <37 weeks compared to women without influenza, but this was not statistically significant after adjustment. There was no significant difference between exposed and unexposed women in the proportion giving birth at <32 weeks. Babies of mothers with influenza were more likely to be admitted to neonatal intensive care (aOR 1·86, 1·01–3·42). Overall, there were few stillbirths, neonatal deaths and congenital abnormalities reported, with no difference observed between babies born to mothers with or without seasonal influenza.

One hundred and six women admitted with influenza (34%) were known to have received an influenza immunization in the relevant season. There was no difference in maternal and perinatal outcomes according to the immunization status of the mother (Table 3).

**Table 3:** Maternal and perinatal outcomes following seasonal influenza in pregnancy outcomes by immunization status.

	Immunization status			OR (95% CI, *P*-value)
	Current (n = 106)	Not current (n = 202)	Unknown (n = 97)	
	Number (%)	Number (%)	Number (%)	
**Maternal Outcomes**				
Admission to ITU	6 (6)	13 (6)	2 (2)	0 ·87 (0·32–2·36, p = 0.788)
Duration of ITU admission (days) Median (IQR)	2.5 (2–3)	3 (2–7)	4.5 (2–7)	
Delivered at time of influenza admission	36 (34)	56 (28)	22 (23)	1·34 (0·81–2·22, p = 0.256)
Pregnancy loss	0 (0)	4 (2)	1 (1)	
Maternal Morbidity	4 (4)	7 (4)	2 (2)	1·06 (0·30–3·72, p = 0.922)
Induction of labour	35 (34)	95 (49)	37 (40)	0·85 (0·64–1·14, p = 0.288)
**Mode of delivery**				
Cesarean overall rate	42 (40)	71 (37)	38 (41)	1·16 (0·71–1·90, p = 0.543)
Emergency cesarean	18 (17)	40 (21)	18 (20)	0·80 (0·43–1·48, p = 0.479)
Assisted vaginal delivery	9 (9)	16 (8)	8 (9)	1·05 (0·45–2·46, p = 0.914)
**Perinatal Outcome**				
Preterm Delivery <37 weeks	16 (15)	21 (11)	15 (16)	1·51 (0·75–3·03, p = 0.251)
Very Preterm Delivery <32 weeks	1 (1)	3 (2)	3 (3)	0·62 (0·06–6·05, p = 0.682)
Live birth	103 (99)	190 (98)	92 (99)	1·63 (0·17–15·8, p = 0.675)
Stillbirth/ Neonatal death	1 (1)	3 (2)	1 (1)	0·61 (0·06–5·99, p = 0.675)
Neonatal unit admission	11 (11)	19 (10)	10 (11)	1·08 (0·49–2·36, p = 0.855)
Congenital abnormality	2 (2)	1 (1)	2 (2)	3·74 (0·34–41·8, p = 0.284)
Low birth weight (<10^th^ centile)	8 (8)	15 (8)	4 (4)	0·99 (0·41–2·43, p = 0.988)
Gestation at delivery (weeks), Median (IQR)	39 (38–40)	39 (38–40)	39 (38–40)	

Based on analysis of data from comparison group women, women that smoked during this pregnancy were less likely to be immunised than non-smokers (aOR 0.42, 95% CI 0.25–0.71). Women with an estimated date of delivery in July to September (n = 66, 9.5%), or October to December (n = 46, 6·6%) were also less likely to be immunised than those expected to deliver in January to March (aOR 0·43, 0·20–0·91; aOR 0·16, 0·04–0·74 respectively) (Table 4). No association was found between maternal age, socioeconomic status, marital status, parity, body mass index or pre-existing medical conditions and current immunization status.

**Table 4:** Characteristics associated with immunization in comparison women.

	Immunization status			OR (95% CI, *P*-value)	aOR (95% CI, *P*-value) ^a^
Independent Variables	Current	Not current	Unknown		
	N = 214	N = 264	N = 216		
	number (%)	number (%)	number (%)		
**Maternal age (years)**					
<20	6 (3)	7 (3)	5 (2)	1·10 (0·36–3·36, p = 0.861)	-
20–35	142 (66)	183 (69)	152 (70)	1·00 (-)	-
≥ 35	66 (31)	74 (28)	59 (27)	1·15 (0·77–1·71, p = 0.483)	-
**Socioeconomic status (woman or partner)**					
Professional	55 (26)	50 (19)	38 (18)	1·38 (0·88–2·18, p = 0.162)	-
Non-professional employed	117 (55)	147 (56)	130 (60)	1·00 (-)	-
Unemployed	20 (9)	35 (13)	21 (10)	0·72 (0·39–1·31, p = 0.280)	-
Unknown	22 (10)	32 (12)	27 (13)		-
**Marital Status**					
Married/Cohabiting	185 (86)	220 (83)	183 (85)	1·00 (-)	-
Single	27 (13)	41 (16)	28 (13)	0·78 (0·46–1·32, p = 0.360)	-
Unknown	2 (1)	3 (1)	5 (2)		
**Smoking Status during pregnancy**					
Did not smoke	184 (86)	197 (75)	168 (78)	1·00 (-)	1·00 (-)
Smoked	28 (13)	64 (24)	43 (20)	0·47 (0·29–0.76, p = 0.002)	0·42 (0·25–0·71, p = 0.001)
Unknown	2 (1)	3 (1)	5 (2)		
**Parity**					
Nulliparous	92 (43)	96 (36)	86 (40)	1·32 (0·91–1·91, p = 0.141)	-
Multiparous	122 (57)	168 (64)	130 (60)	1·00 (-)	-
**Body Mass Index**					
Normal or overweight (<30)	162 (76)	193 (73)	163 (75)	1·00 (-)	-
Obese (≥30)	48 (22)	59 (22)	48 (22)	0·97 (0·63–1·50, p = 0.888)	-
Unknown	4 (2)	12 (5)	5 (2)		
**Multiple Pregnancy**					
Yes	8 (4)	3 (1)	2 (1)	1·00 (-)	-
No	206 (96)	261 (99)	214 (99)	3·38 (0·89–12·90, p = 0.075)	-
**Relevant pre-existing medical condition**					
Yes	25 (12)	32 (12)	20 (9)	0·95 (0.55–1.67, p = 0.871)	-
No	189 (88)	231 (88)	195 (90)	1·00 (-)	-
Unknown	0 (0)	1 (0)	1 (0)		
**Asthma**					
Yes	10 (5)	14 (5)	6 (3)	0·87 (0·38–2.00, p = 0.747)	-
No	204 (95)	249 (94)	210 (97)	1·00 (-)	-
Unknown	0 (0)	1 (0)	0 (0)		
**Known previous pregnancy complications**^**b**^					
Yes	9 (4)	10 (4)	16 (7)	1·12 (0·44–2·80, 0.816)	-
No	205 (96)	254 (96)	200 (93)	1·00 (-)	-
**Estimated date of delivery**					
Jan–Mar	129 (60)	138 (52)	102 (47)	1·00 (-)	1·00 (-)
Apr–Jun	69 (32)	70 (27)	74 (34)	1·05 (0·70–1·59, 0.800)	1·00 (0·64–1·56, p = 0.996)
Jul–Sep	13 (6)	33 (13)	20 (9)	0·42 (0·21–0·84, 0.013)	0·43 (0·20–0·91, p = 0.028)
Oct–Dec	3 (1)	23 (9)	20 (9)	0·14 (0·04–0·48, 0.002)	0·16 (0·04–0·74, p = 0.018)

^a^ OR adjusted for smoking status and estimated date of delivery.

^b^Previous pregnancy complications include mental health problems, diabetes, hypertensive disorders and pre-existing medical conditions exacerbated by pregnancy together with mid-trimester loss, stillbirth, and pre-term labour or birth.

## Discussion

### Main findings

We observed an incidence of hospital admission with seasonal influenza in pregnancy of 2.7/10,000 maternities, which is in keeping with hospitalisation rates reported for pandemic influenza in pregnancy. Our main findings showed that women with influenza were less likely to have been immunised in the relevant season and less likely to be professionally employed than women the comparison group of women without influenza. They had a 2·5-fold increased odds of having had a previous severe pregnancy complication (e.g. hypertensive disorders of pregnancy) or asthma than comparison women. Women with influenza and their babies were more likely to be admitted to intensive care, and to have a cesarean birth compared to the unexposed group. Women that smoke in pregnancy and women that are due to give birth between July and December were less likely to be immunised against influenza. Immunization prior to seasonal influenza was not associated with any significant difference in maternal or perinatal outcomes.

### Strengths and limitations

To our knowledge, this is the first national, prospective population‐based study on hospital admission with seasonal influenza in pregnancy. The main strengths of this study are the robust method of nationwide prospective identification of exposed women. This allows reporting of population‐based data on the causal agents and outcomes of this relatively uncommon condition. Despite this, the number of severe outcomes in this study are small leading to wide confidence intervals for these analyses. Inclusion of laboratory confirmed influenza requiring hospital admission means that only true cases were included, but we are unable to evaluate the outcomes of mild cases or those that did not present to hospital; therefore, the true population incidence may be greater than reported. The comparison to a contemporaneous unexposed group allowed conclusions to be drawn on the characteristics associated with hospitalisation with influenza and immunization and the use of comparison women from the same centres as women with influenza means there is low risk of selection or measurement bias.

A further limitation is that the time period in which this study was undertaken included a period where the influenza vaccine was less effective than previous years [32]. This may have influenced the incidence of influenza in immunised individuals as well as the risk of maternal and perinatal morbidity in this group. In addition, we have shown that the likelihood of being immunised differs according to your estimated date of delivery and thus the trimester of pregnancy you are in at the time that immunization programmes are widely available, however access to immunization likely differs within these groups. Some data were incomplete, the most frequent missing data was immunization status (missing for 24% of women with influenza and 31% of comparison women), as it is not routinely captured electronically in all hospitals.

### Interpretation

Prior to this study there was an absence of high-quality studies reporting maternal and perinatal outcomes following seasonal influenza [11, 20]. This study provides robust evidence that women hospitalised with influenza and their babies are more likely to be admitted to intensive care, and to have a cesarean birth.

The high proportion of cesarean births observed in this study were also reflected during the H1N1 pandemic, where 58% of pregnant women had a cesarean birth compared to a baseline rate of 31% [9]. During the SARS-CoV2 pandemic we have also seen elevated cesarean section rates reported globally [40]. This has substantial health and economic impacts, and further research is required to explore the implications of this and how to prioritize services in future pandemic situations.

We also showed that immunization is effective at preventing influenza in pregnancy, however the overall uptake of influenza immunization in this cohort was low (26% in women with seasonal influenza and 31% of comparison group), even compared to the national average of 45% in 2018–19. Increasing uptake of immunization in pregnancy may therefore reduce this avoidable morbidity. This is particularly important in the context of services pressurised by SARS-CoV-2 infection.

The reasons for variable uptake of vaccinations in pregnancy are complex and interrelated and dependent on the setting. For example, in the UK operational issues such as cold-chain storage and procurement are likely less of a challenge than in low-and-middle resourced countries. The proportion of pregnant women that have been reported to refuse or decline immunization in the UK is small (5·7%) [32]. This suggests that many more women are not offered or advised to receive immunization, and therefore have no opportunity to decline [33]. Indeed, it is known that there is a strong link between provision of information on immunization by maternity healthcare workers and vaccine uptake in women [41–43]. A survey of 3441 practice nurses, midwives and health visitors working in England found that, whilst the majority stated they routinely recommend influenza immunization in pregnancy (73%), fewer were immunised themselves (58% of midwives, 61% of health visitors and 79% of practice nurses), most commonly citing concern over side effects. Whilst midwives were identified as having the main responsibility for advising on immunization, only 62% had received training, 60% were confident in giving advice and 9% reported providing immunization to pregnant women, even though the majority would be happy to do so [44]. There may be a role for steps to increase healthcare professionals’ awareness about the effectiveness and safety of immunization in pregnancy and increase provision within antenatal care settings to further prevent avoidable morbidity.

The majority of studies to date around the characteristics associated with severe outcomes from influenza in pregnancy were undertaken during the 2009 H1N1 pandemic [11, 45]. We provide evidence from a national cohort on the characteristics and outcomes associated with seasonal influenza in relation to immunization status. We have shown that women due to give birth between October and December likely face a greater risk of developing influenza in pregnancy, yet they are less likely to be immunised. The main immunization programme in the UK usually commences in November, when this group are in their third trimester. Even in their third trimester, women should be encouraged to receive immunization at the earliest opportunity. Women due to deliver between July and September were also less likely to be immunised. This group would be in their first trimester during the influenza period; therefore, this may represent the delay in recognising or booking pregnancy with a health care professional or an intentional delay through concern over the safety of immunization in the first trimester. Women should be reassured that immunization against influenza is safe even in early pregnancy [22] and immunization should be encouraged at any point during the seasonal influenza period.

Although this study did not show increased risk of hospital admission with influenza in women that smoke, this group were less likely to be immunised and therefore were at greater risk of developing influenza. It is plausible that influenza may be associated with more severe respiratory complications in this group, who as a result should be a specific focus of immunization programmes. Hence this study highlights three high-risk groups for whom there should be a greater focus on facilitating immunization. Whilst there is considerable evidence about the barriers to immunization in pregnancy, there is little evidence to support interventions to overcome them [46], and this warrants further research.

Previous studies have demonstrated a reduced risk of neonatal complications following influenza in women that were immunised [21–24], whereas we did not show any significant difference in maternal or perinatal outcomes. It is possible that our study is underpowered to show a meaningful difference in this subgroup analysis. Further research is required to explore this finding.

## Conclusions

Hospitalisation with seasonal influenza in pregnancy is not uncommon and is associated with increased risk of morbidity for both the mother and baby. We showed that influenza immunization reduces the risk of hospitalisation with influenza in pregnancy. However, although immunization against influenza is known to be safe, uptake of influenza immunization in the UK remains low. There is a need to increase awareness of safety and effectiveness of immunization in pregnancy and provision within antenatal care settings, especially for high-risk groups.
